# Odontological Society of Great Britain

**Published:** 1884-09

**Authors:** 


### Odontological Society of Great Britain.

Mr. J. S. Turner, M.R.C.S., L.D.S., President, in the chair. Mr. Storer Bennett showed, for Mr. Balkwill, of Plymouth, the skull of an English black rat, which illustrated in a very remarkable manner the serious results which might happen to rodent animals from the continuous growth of their characteristic teeth when from any cause their antagonism was lost. In this case the two lower incisors had been deflected to the left, and the two upper to the right, so that they did not meet in the usual way. The right upper tooth had described one complete circle and three-fourths of another, but, being well inclined outwards, it had not met with any obstacle. The right lower tooth had penetrated the upper jaw and the parts above, and its tip projected a quarter of an inch above the highest point of the skull. The left upper tooth, after describing a complete circle, had encountered the front of the upper jaw, which it had penetrated to the depth of one-third of an inch. The left lower tooth had been broken off. The lower jaw was thus quite fixed, and the animal could only have obtained nourishment by means of suction.

Mr. E. G. Betts stated that he had found ethylate of sodium very useful for obtunding pain of sensitive dentine. Before applying it, the dentine should be carefully dried, and the application could be repeated at intervals as the sensibility returned. As it was used by the medical profession for destroying cutaneous nævi, he thought it might be useful in destroying the tooth pulp; but in this he had been disappointed.

Mr. J. H. Mummery read notes of a case of extensive alveolar abscess of obscure origin. The patient, a strong, healthy-looking young man from Natal, had suffered more than a year from constant purulent discharge from on opening below the angle of the jaw on the right side, which was a source of great annoyance to him. He had consulted several surgeons, one of whom sent him to Mr. Mummery with a request that he would remove the necrosed roots of the right lower first molar. This was done, but did not affect the abscess. The second molar had a small cement filling, but was, to all appearances, healthy, as was also the third molar. Six months later the patient returned in the same condition, and now consented to a further examination of the second molar, which was, however still firm, and free from tenderness or percussion. On drilling into the pulp cavity, pus escaped; the tooth was at once extracted, and the sinus healed within a week, and a complete cure effected. The patient stated that the tooth had cost him between £400 and £500 in traveling expenses and surgical fees.

Mr. Betts then read a paper entitled "Observations on the Teeth of Certain Rodents," in which he disproved the statement of Professor Hilgendorff, since copied into other works, that the incisors of the Leporidæ had a complete investment of enamel, and showed that the thin, transparent layer at the back of the incisors of hares and rabbits were really continued from the cementum, and could clearly be demonstrated not to be enamel. Although apparently a small matter, it was of more importance than appeared at first sight, since this supposed fact had been held to show that the Leporidæ were in this respect a missing link between the rodents and other mammalia, whilst in reality the exact relations of this large class to the rest of the animal kingdom must still be considered an open problem.

The result of Mr. Bett's observations was confirmed by Messrs. A. Underwood and Chas. Tomes, who also commented on the obscure zoological relations of the rodent class, and the latter suggested that some of the missing links might yet be supplied by a careful study of the marsupials.—*Medical Press.*

---

THE judgment which was rendered over a year ago, in New York, by Justice Van Vorst, at Supreme Court, special term, against the "United States (Eclectic) Medical College," depriving the institution of its charter, was affirmed in May, 1883. From that judgment the defendants appealed to the Court of Appeals, and the latter has just handed down a decision affirming the judgment. As the attorneys for the "Buffalo College of Physicians and Surgeons" stipulated to let the case against that school abide the event of this suit, the decision disposes of both of these bogus colleges.

